# Determination of individual contact interfaces in carbon nanotube network-based transistors

**DOI:** 10.1038/s41598-017-05653-x

**Published:** 2017-07-14

**Authors:** Jinsu Yoon, Meehyun Lim, Bongsik Choi, Dong Myong Kim, Dae Hwan Kim, Sungho Kim, Sung-Jin Choi

**Affiliations:** 1 School of Electrical Engineering, Kookmin University, Seoul, 02707 Korea; 2 Mechatronics R&D Center, Samsung Electronics, Gyeonggi-do, 18448 Korea; 3 Department of Electrical Engineering, Sejong University, Seoul, 05006 Korea

## Abstract

Carbon nanotubes (CNTs) used as semiconducting channels induce high mobility, thermal conductivity, mechanical flexibility, and chemical stability in field-effect, thin-film transistors (TFTs). However, the contact interfaces in CNT-TFTs have contact resistances that are difficult to reduce; this contact resistance can eventually limit the overall performance of CNT-TFTs. The contact interface between the source/drain electrodes and CNTs, especially for those CNT-TFTs in which the channel comprises randomly networked CNTs, plays a particularly dominant role in determining the performance and degree of variability in CNT-TFTs. However, no studies have reported a determination method that individually extracts each contact resistance at the source/drain electrodes. The present work presents an efficient method for directly determining the contact interfaces in CNT-TFTs by extracting each contact resistance produced at the source (*R*_*S*_) and drain (*R*_*D*_) electrodes. Moreover, we comprehensively simulated the randomly networked CNTs using an in-depth Monte-Carlo method, which provides an efficient method for visualizing the uniformity of a CNT network with various controllable CNT parameters. The proposed method provides guidance and a means for optimizing the design of the CNT network channel in CNT-TFTs and additional insights into improving the performance of CNT-TFTs.

## Introduction

Carbon nanotubes (CNTs) have been intensely studied for use as channel materials in thin-film transistors (TFTs) due to their high carrier mobility, excellent chemical stability, mechanical flexibility, and compatibility with solution-based processing^1–11^. Example applications of CNT-TFTs include simple logic circuits^7, 8^, display electronics^2, 3^, sensors^7^, and radio frequency identification antennae^1, 7^. However, research regarding CNT-based TFTs, i.e., CNT-TFTs, shares one critical drawback: problems arising from the co-existence of both semiconducting and metallic CNTs during their growth. The unwanted metallic CNTs, particularly in digital logic applications, will correspondingly lead to low on/off current ratios resulting from a high off-state leakage current and high subthreshold swing^12, 13^. Therefore, the difficulty in achieving high-purity semiconducting CNTs during synthesis has led to extensive efforts in solution-process-based purification using techniques such as density gradient ultracentrifugation^14, 15^, selective polymer wrapping^16^, chromatography^17, 18^, and aqueous two-phase separation^19^, which allow CNTs to be rapidly and inexpensively separated based on their electronic types, i.e., semiconducting and metallic CNTs.

Despite these efforts, there are still factors limiting the ultimate performance of CNT-TFTs, such as the contact interface, as quantified by the contact resistance, which is difficult to reduce, even in the ballistic transport regime for application to logic devices^20–22^. Therefore, the contact interface plays a particularly dominant role in determining the performance and degree of variability in CNT-TFTs and should thus be further optimized and investigated in depth. In particular, for the case of CNT-TFTs in which the channel is composed of randomly networked CNTs, the appearance of asymmetric contact interfaces, i.e., different contact resistance at the source (*R*_*S*_) and drain (*R*_*D*_) regions, at the source and drain (S/D) regions is unavoidable due to non-uniformly networked CNTs in the channel. Thus, an effective and efficient method to individually quantify the contact interfaces at each S/D of CNT-TFTs, i.e., direct measurement to separately quantify the contact resistance at the S/D regions in the CNT-TFTs, should be developed to guide the further optimization of the performances of CNT-TFTs.

In this work, we present a simple technique for quantifying the individual contact resistance, i.e., *R*_*S*_ and *R*_*D*_, in CNT-TFTs constructed from a solution-processed, highly purified 99% semiconducting CNT solution. By exchanging the location of the ground node between S/D electrodes in the CNT-TFTs during the measurement of transfer characteristics, we could quantify the contact interface by separately extracting the values of *R*_*S*_ and *R*_*D*_. Our proposed method was applied to quantify the values of *R*_*S*_ and *R*_*D*_ in CNT-TFTs for various densities of CNTs in the network channel; this method enables a comprehensive investigation of the performance of CNT-TFTs. The network channel with higher CNT density showed more balanced values of *R*_*S*_ and *R*_*D*_, i.e., approaching an identical value between them, than the network channel with a lower CNT density. This observation was confirmed by a detailed Monte-Carlo simulation. The simulation results indicate that a higher density and longer length of CNTs lead to balanced performance at the S/D electrodes. Our finding can be easily understood and is straightforward, but to the best of our knowledge, this type of research has not yet been performed, despite the impact of the contact interface being critical with respect to device performance. Therefore, we believe that our developed technique and simulation in this work would be fruitful for predicting CNT-TFT circuit performance, particularly in the ultimately scaled CNT-TFT, as well as for the design of the device layout and fabrication process.

## Results and Discussion

**Figure 1:**
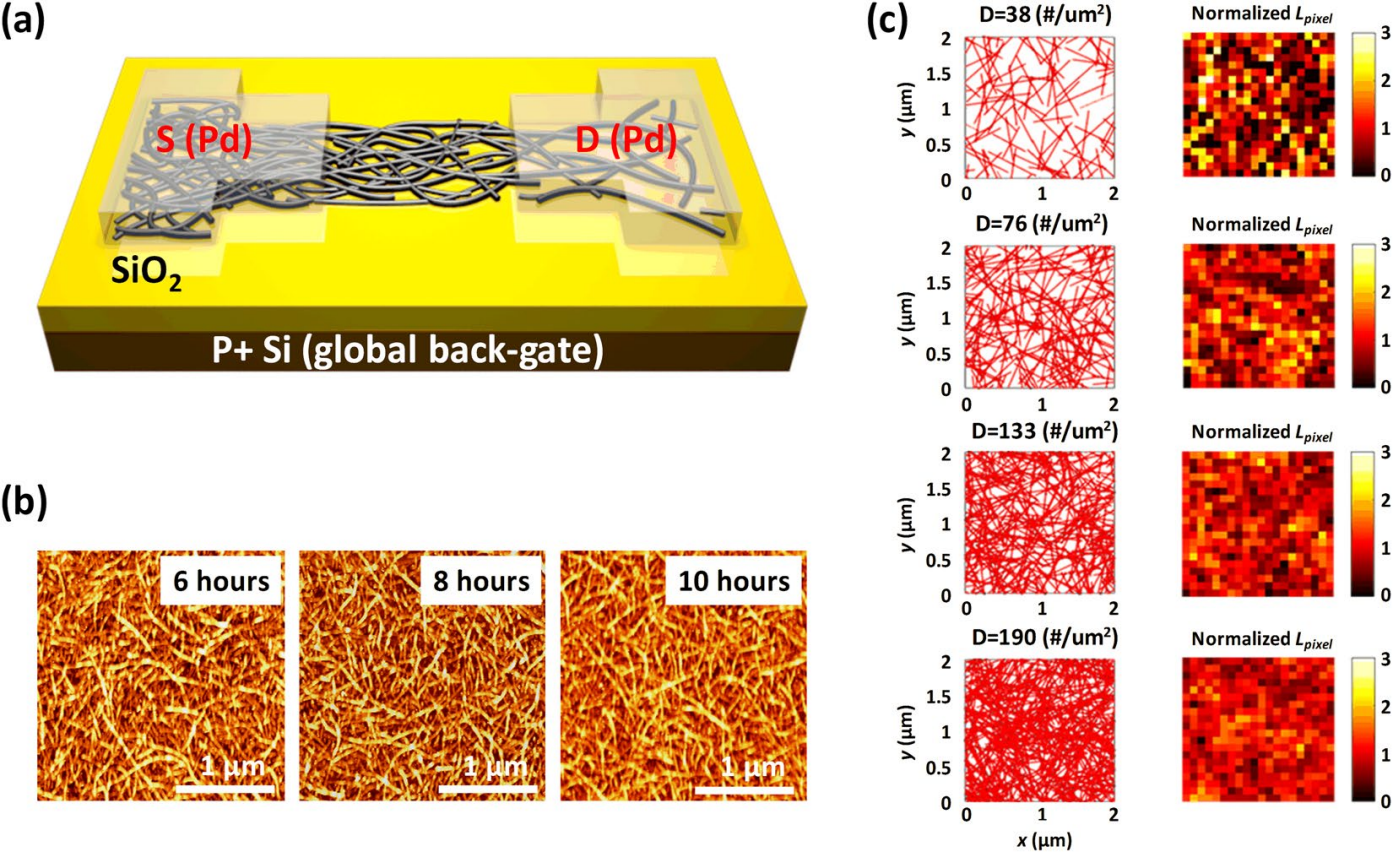
(**a**) Schematic of the CNT-TFT used in this work. (**b**) AFM image (2.5 × 2.5 µm, z-scale is 10 nm) of the CNT network channel constructed from a 99% semiconducting CNT solution with deposition times of 6, 8, and 10 h. (**c**) Simulation results of the randomly generated CNT network with different CNT densities (left) and visualized length (*L*_*pixel*_) of CNTs for each 400 pixels normalized by the average value of *L*_*pixel*_ from all pixels.

Highly purified 99% semiconducting CNT solution (purchased from NanoIntegris, Inc.) was used to create the randomly networked CNT channel. The device structure of the CNT-TFT in this work is illustrated in Fig. 1a. It incorporated a global back-gate, a highly purified and pre-separated 99% semiconducting CNT network channel, a SiO_2_ gate dielectric, and palladium (Pd) S/D electrodes. The fabrication of CNT-TFTs started from the silicon wafer, which was highly p-doped to serve as the global back-gate, with a thermally grown 55-nm-thick silicon dioxide (SiO_2_) layer. The randomly networked semiconducting CNT networks were obtained by functionalizing the SiO_2_ surface with an amine group using 0.1 g/mL poly-L-lysine solution, which acts as an effective adhesion layer to hold the CNTs^8, 23, 24^. The substrate was then rinsed with deionized (DI) water. Subsequently, the CNT network channel was formed by immersing the chip into a 99% semiconducting enriched CNT solution (0.01 mg/ml) for three different times of 6, 8, and 10 h, followed by thorough rinsing with isopropanol and DI water. The density of the CNTs in the network can be controlled by adjusting the deposition time, and thus, the electrical parameters of the fabricated CNT-TFTs with various densities of CNTs could be significantly altered (as will be discussed). The atomic force microscopy (AFM) images shown in Fig. 1b indicate an increase in CNT network density with deposition time. The CNT density was almost saturated with increasing deposition time; hence, a noticeable increase in CNT density not apparent to the naked eye. However, the average CNT densities at each deposition time were carefully calculated manually as 63.8 ± 6.4, 81.4 ± 4.8, and 94.5 ± 3.3 tubes/µm^2^ at 6, 8, and 10 h, respectively. In particular, CNTs with high semiconducting purity (99% in our work) were short compared to the CNTs with lower purity; thus, a longer deposition time was required to form a well-percolated CNT network channel^2, 12, 23^. Furthermore, the AFM images confirmed that the standard deviation of the CNT densities in the different spots of the network decreased with longer deposition time. This observation was also confirmed by our comprehensive Monte-Carlo simulation (for details of the simulation method, see the Supplementary Information, Fig. S1), as shown in Fig. 1c. We visualized, i.e., digitized, the uniformity image of the CNT network that was randomly generated using the random number generator function in Matlab, as shown in Fig. 1c. In the simulation, the entire CNT network with a size of 2 µm × 2 µm was divided into a large number of small pixels with a size of 0.1 µm × 0.1 µm. To visualize and quantify the uniformity of the CNT network with different densities of CNTs, the total length of CNTs in each pixel (*L*_*pixel*_) was calculated and normalized with the averaged value of *L*_*pixel*_ calculated from all pixels of the entire CNT network (for other visualizations of the uniformity of the CNT network, see the Supplementary Information, Fig. S2). Based on the simulated results, it was anticipated that a network with a higher density of CNTs would lead to better performance of the CNT-TFTs regarding the contact interfaces because the higher density of CNTs provides a large and uniform contact area between CNTs and the metal S/D electrodes. Importantly, our developed simulation enables the network to be predicted and optimized using various physical parameters of CNTs to achieve superior CNT-TFT performance, as will be discussed below.

After the formation of the CNT network channels, to form the S/D electrodes, Ti/Pd (2 nm/40 nm) layers were sequentially deposited and patterned using thermal evaporation and a lift-off process. Finally, the channel area was defined by an additional photolithography step and oxygen plasma etching. This step also removes undesirable leakage current paths outside of the designated channel region. The values of the channel width (*W*) and length (*L*) of the fabricated CNT-TFTs are 2, 3, 5, and 30 µm and 1.4, 2.2, and 3 µm, respectively.

**Figure 2:**
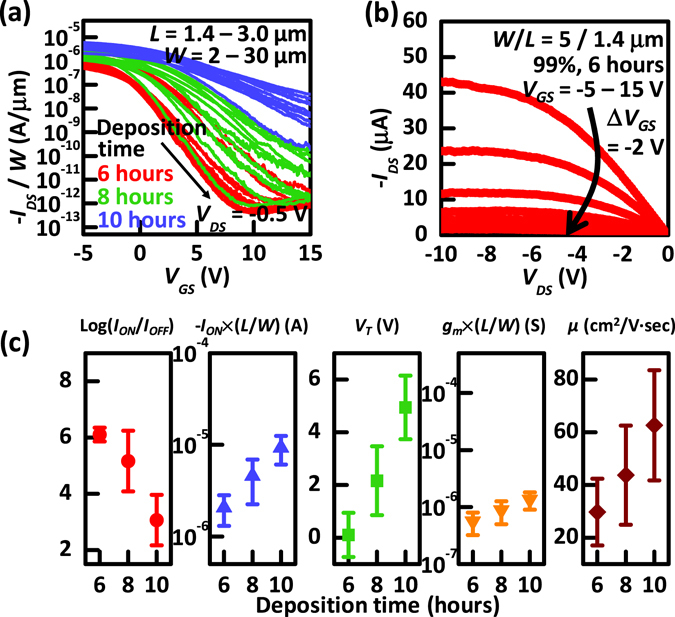
(**a**) Transfer characteristics (*I*_*DS*_-*V*_*GS*_) of three sets of CNT-TFTs produced for different CNT densities in the network channel. (**b**) Output characteristics (*I*_*DS*_-*V*_*DS*_) of a CNT-TFT fabricated from a deposition time of 6 h. (**c**) Log(*I*_*ON*_/*I*_*OFF*_), normalized on-state current (*I*_*ON*_), threshold voltage (*V*_*T*_), normalized transconductance (*g*_*m*_), and mobility (*µ*) for CNT-TFTs with different densities of CNTs.

**Figure 3:**
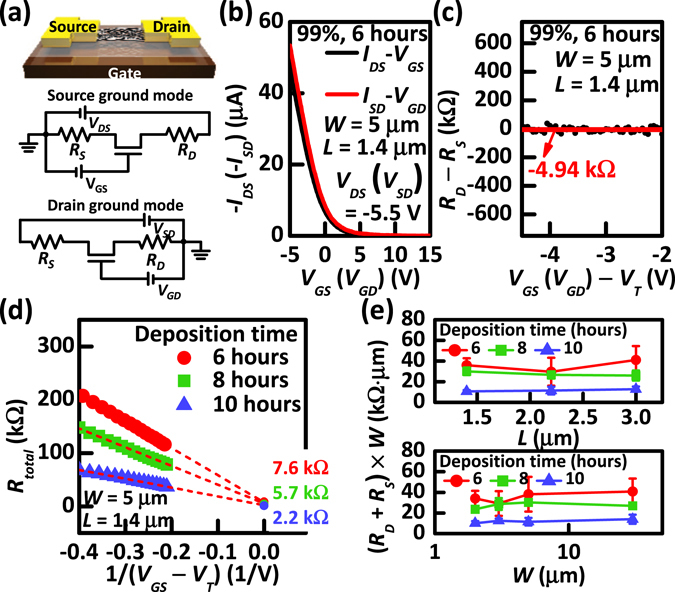
(**a**) Schematic illustration and equivalent circuits with *R*_*S*_ and *R*_*D*_ of the source and drain ground mode for CNT-TFTs. (**b**) Measured transfer characteristics for the source and drain ground mode. (**c**) The difference of *R*_*D*_ and *R*_*S*_, i.e., *R*_*D*_ − *R*_*S*_, calculated from two transconductances (*g*_*mS*_ and *g*_*mD*_) obtained from the measured transfer characteristics in the source and drain ground mode. (**d**) *R*_*total*_ vs. 1/(*V*_*GS*_ − *V*_*T*_) for the channel resistance method (CRM). The combined contact resistance, i.e., sum of *R*_*D*_ and *R*_*S*_, is extracted as 7.6, 5.7, and 2.2 kΩ at 1/(*V*_*GS*_ − *V*_*T*_) = 0 with different densities of CNTs in the network. (**e**) Normalized *R*_*D*_ + *R*_*S*_ vs. *L* and *W* for CNT-TFTs with different densities of CNTs in the network.

First, we compared the transfer characteristics, i.e., drain current (*I*_*DS*_)-gate voltage (*V*_*GS*_), produced from the 99% semiconducting solution at a drain voltage (*V*_*DS*_) of −0.5 V according to the different CNT network densities, as shown in Fig. 2a. As the CNT network densities were increased, the on-state performance, i.e., on-state current (*I*_*ON*_), defined at *V*_*GS*_ = −5 V and *V*_*DS*_ = −0.5 V, also increased. In contrast, the on/off current ratio, i.e., *I*_*ON*_/*I*_*OFF*_ (the off-state current, *I*_*OFF*_, is defined at *V*_*GS*_ = 15 V and *V*_*DS*_ = −0.5 V), exhibits the opposite trend, decreasing with increasing CNT network density. This trend always occurs because the probability of a metallic interconnection between the S/D electrodes increases at higher CNT network densities. This observed trend is consistent with prior reports and suggests that CNT-TFTs exhibit an inherent trade-off between *I*_*ON*_ and *I*_*ON*_/*I*_*OFF*_. However, this trend is likely to be alleviated for a higher purity of semiconducting CNTs (i.e., above 99% semiconducting purity); hence, excellent electrical performance is expected to be achieved with simultaneously high *I*_*ON*_ and *I*_*ON*_/*I*_*OFF*_. The representative output characteristics, i.e., *I*_*DS*_-*V*_*DS*_, of the CNT-TFTs at a deposition time of 6 h (i.e., average CNT density of 63.8 tubes/µm^2^) are also shown in Fig. 2b. The curve appears to be linear at small *V*_*DS*_ values, indicating that ohmic contacts are well formed between the Pd S/D electrodes and CNTs. At more negative values of *V*_*DS*_, the device exhibits saturation behaviour, indicating reasonable field-effect operation. The key device performance metrics, such as log (*I*_*ON*_/*I*_*OFF*_), normalized on-state current (−*I*_*ON*_ × *L*/*W*), threshold voltage (*V*_*T*_), normalized transconductance (*g*_*m*_ × *L*/*W*), and field-effect mobility (*µ*), are summarized in Fig. 2c to directly investigate the electrical performances of a total of 12 CNT-TFTs with various CNT network densities. The device mobility was calculated from the following equation:

1 $$\mu =\frac{L}{W}\frac{1}{{C}_{G}{V}_{DS}}{g}_{m}$$

where *g*_*m*_ is the transconductance and *C*_*G*_ is the gate capacitance per unit area. The gate capacitance is calculated using a more rigorous and sophisticated model than the parallel-plate model^8, 12, 25^, as given below:

2 $${C}_{G}={\{{C}_{Q}^{-1}+\frac{1}{2\pi {\varepsilon }_{{0}}{\varepsilon }_{ox}}\mathrm{ln}[\frac{{{\Lambda }}_{{0}}}{R}\frac{\sinh (2\pi {t}_{ox}/{{\Lambda }}_{{0}})}{\pi }]\}}^{-1}{{\Lambda }}_{{0}}^{-1}$$

where 1/*Λ*_*0*_ denotes the linear density of CNTs in the network, which was approximately extracted as 14, 16, and 19 tubes/µm for deposition times of 6, 8, and 10 h, respectively), *C*_*Q*_ = 4.0 × 10^−10^ F/m is the quantum capacitance of a single CNT^26^, *t*_*ox*_ = 55 nm is the thickness of the SiO_2_ dielectric layer, *R* = 0.7 nm is the radius of the CNTs, and *ε*_*0*_ *ε*_*ox*_ = 3.9 × 8.854 × 10^−14^ F/cm is the dielectric constant. As noted above, the most noticeable feature from the results is the trade-off relationship between *I*_*ON*_ and *I*_*ON*_/*I*_*OFF*_, implying that the CNT network densities would be adjusted according to the different target applications. In other words, a low CNT network density can be used for compliant digital electronics or as switches in a display backplane, whereas a high CNT network density is ideal for high-frequency applications^2, 8, 12, 27^. We also summarized other important device performance metrics according to different *L* values of the fabricated CNT-TFTs with deposition time (6, 8, and 10 h) in the Supplementary Information, Fig. S3. Using the fabricated CNT-TFTs with various densities of CNTs in the network, we could quantify the contact interfaces by extracting the individual *R*_*S*_ and *R*_*D*_ in the CNT-TFTs using our proposed method detailed below. The developed model was based on the construction of the equivalent circuit of CNT-TFTs with finite, non-zero *R*_*S*_ and *R*_*D*_, as shown in Fig. 3a. These finite contact resistances result in unwanted potential drops that depend on the magnitude of the contact resistances; hence, the effective voltages, such as *V*_*GS*_ and *V*_*DS*_, become reduced according to their values. First, we measured the typical transfer characteristics of the CNT-TFTs at various densities of CNTs in the network. By exchanging the location of the ground nodes between the S/D electrodes (that is, one is the ground node connected at the source electrode (named the ‘source ground mode’), and the other is the ground node connected at the drain electrode (named the ‘drain ground mode’)), *I*_*DS*_-*V*_*GS*_ and *I*_*SD*_-*V*_*GD*_ curves were obtained from the source ground mode and drain ground mode, as shown in Fig. 3b. Notably, the curves do not appear to be entirely identical; this observation is a particularly important clue with regard to the extraction of individual contact resistances of *R*_*S*_ and *R*_*D*_ values.

Considering the contact resistances, the intrinsic gate-to-source voltage (*V*_*GS,int*_), i.e., the pure potential difference induced between the gate and source electrodes without a voltage drop arising from the contact resistance, can be expressed as *V*_*GS,int*_ = *V*_*GS*_ − *I*_*DS*_ *R*_*S*_ for the source ground mode and as *V*_*GD,int*_ = *V*_*GD*_ − *I*_*SD*_ *R*_*D*_ for the drain ground mode. Thus, the saturation current for the source ground mode in Fig. 3b can be modelled as

3 $${I}_{DS}=\frac{{\mu }{C}_{G}}{2}(\frac{W}{L}){({V}_{GS}-{I}_{DS}{R}_{S}-{V}_{T})}^{2}$$

where *V*_*T*_ is the threshold voltage. Considering voltage drops across *R*_*S*_ under the saturation region, the extrinsic (*g*_*mS*_) and intrinsic (*g*_*mS,int*_) transconductance values as a function of *V*_*GS*_ are obtained through the equations below.

4 $${g}_{mS}({{V}}_{GS})=\frac{\partial {I}_{DS}}{\partial {V}_{GS}}={\mu }{C}_{G}(\frac{W}{L})\times ({V}_{GS}-{I}_{DS}{R}_{S}-{V}_{T})(1-\frac{\partial {I}_{DS}}{\partial {V}_{GS}}{R}_{S})$$

5 $${g}_{mS,{\rm{int}}}({{V}}_{GS})=\frac{\partial {I}_{DS}}{\partial {V}_{GS,{\rm{i}}{\rm{n}}{\rm{t}}}}={\mu }{C}_{G}(\frac{W}{L})\times ({V}_{GS}-{I}_{DS}{R}_{S}-{V}_{T})$$

These equations provide a means to relate *g*_*mS,int*_ to *g*_*mS*_ as follows:

6 $${g}_{mS,\mathrm{int}}=\frac{{g}_{mS}}{(1-{g}_{mS}{R}_{S})}$$

Moreover, the relation of *g*_*mD,int*_ and *g*_*mD*_ is obtained using the same method as the saturation current for the drain ground mode below.

7 $${g}_{mD,\mathrm{int}}=\frac{{g}_{mD}}{(1-{g}_{mD}{R}_{D})}$$

The intrinsic transconductance values, such as *g*_*mS,int*_ and *g*_*mD,int*_, under the saturation regime are essentially identical for both the source and drain ground mode configurations because the asymmetric effects of *R*_*S*_ and *R*_*D*_ were already included in the extrinsic transconductance values, i.e., *g*_*mS*_ and *g*_*mD*_. Hence, based on the equal property of *g*_*mS,int*_ and *g*_*mD,int*_, the difference between *R*_*D*_ and *R*_*S*_, i.e., *R*_*D*_ − *R*_*S*_, can be experimentally obtained from the measurements of *g*_*mS*_ and *g*_*mD*_ below.

8 $${R}_{D}-{R}_{S}=\frac{{g}_{mS}-{g}_{mD}}{{g}_{mD}\,{g}_{mS}}$$

This is the highlighted idea in our proposed method for quantifying the individual contact resistances of *R*_*S*_ and *R*_*D*_ in CNT-TFTs.

Next, the combined contact resistance associated with the sum of *R*_*D*_ and *R*_*S*_, i.e., *R*_*D*_ + *R*_*S*_, can be obtained below. Under the linear operating regime with small *V*_*DS*_, the *I*_*DS*_ can be approximated as shown below, with consideration of the intrinsic drain-to-source voltage, i.e., *V*_*DS,int*_ = *V*_*DS*_ − *I*_*DS*_(*R*_*D*_ + *R*_*S*_),

9 $${I}_{DS}=\frac{\mu {C}_{G}W}{L}[({V}_{GS}-{V}_{T})\times ({V}_{DS}-{I}_{DS}\,({R}_{D}+{R}_{S}))]$$

Therefore, the total resistance (*R*_*total*_) can be summarized as

10 $${R}_{total}({{V}}_{GS})=\frac{{V}_{DS}}{{I}_{DS}}={R}_{D}+{R}_{S}+\frac{L}{\mu {C}_{G}W({V}_{GS}-{V}_{T})}={R}_{D}+{R}_{S}+L\times {r}_{ch}$$

where *r*_*ch*_ is a *V*_*GS*_-dependent channel resistivity. Therefore, the sum of *R*_*D*_ and *R*_*S*_ can be experimentally extracted by the channel resistance method (CRM) though the equation

11 $${\frac{{V}_{DS}}{{I}_{DS}}|}_{{\rm{extrapolated}}{\rm{to}}\frac{1}{{(V}_{{\rm{GS}}}{-V}_{{\rm{T}}})}\to {\rm{0}}}={R}_{D}+{R}_{S}$$

which makes the *V*_*GS*_-dependent component (*L* × *r*_*ch*_) negligible because of the highly conductive channel at *V*_*GS*_ → ∞ (1/(*V*_*GS*_ − *V*_*T*_) ≈ 0)^28^. Using the CRM, we obtained *R*_*D*_ + *R*_*S*_ for every CNT-TFT, which enabled an accurate determination of each contact interface in all CNT-TFTs. By contrast, using the transmission line model (TLM), only one value of *R*_*D*_ + *R*_*S*_ can be obtained from CNT-TFTs with different *L* values, which hinders the delicate analysis, especially for CNT-TFTs in which large variations of electrical properties exist due to randomly networked CNTs in the channel, although the effect of CNT-CNT junction resistance (*R*_*J*_) can be negligible using the TLM. Moreover, it is been reported that *R*_*J*_ can also be strongly dependent on *V*_*GS*_^29^. We found that the values of *R*_*D*_ + *R*_*S*_ extracted using the CRM and TLM were very similar (for details of the extraction methods for *R*_*D*_ + *R*_*S*_, see the Supplementary Information, Fig. S4). Combining the component of (*R*_*D*_ − *R*_*S*_) obtained from the transconductance values in the S/D ground mode and the component of (*R*_*D*_ + *R*_*S*_) from the CRM result, we finally quantify each contact interface in the CNT-TFT using the equation below.

12 $${R}_{D}=[({R}_{D}-{R}_{S})+({R}_{D}+{R}_{S})]/2,\,{R}_{S}=-[({R}_{D}-{R}_{S})-({R}_{D}+{R}_{S})]/2$$

To verify our proposed method for the contact interfaces in the CNT-TFTs, we applied the method to the CNT-TFTs for various CNT densities in the network. First, for *R*_*D*_ − *R*_*S*_, we measured the transconductance values, i.e., *g*_*mS*_ and *g*_*mD*_, from two transfer characteristics, i.e., *I*_*DS*_−*V*_*GS*_ (*I*_*SD*_−*V*_*GD*_), of the CNT-TFTs measured in the source ground mode (drain ground mode) with a high drain voltage for the saturation operating regime at *V*_*DS*_ (*V*_*SD*_) = −5.5 V, as shown in Fig. 3b. Next, *R*_*D*_ − *R*_*S*_ was experimentally obtained using Eq. (8), as shown in Fig. 3c. The red line indicates the average value of the extracted *R*_*D*_ − *R*_*S*_ in saturation regime (|*V*_*GS*_(*V*_*GD*_) − *V*_*T*_| < |*V*_*DS*_ (*V*_*SD*_)|). Moreover, the *V*_*GS*_-dependent *R*_*total*_ values obtained from *V*_*DS*_/*I*_*DS*_ for different CNT network densities were then measured to obtain the *R*_*D*_ + *R*_*S*_ value, as shown in Fig. 3d. Through the CRM, i.e., Eqs (10) and (11), the value of *R*_*D*_ + *R*_*S*_ can be extracted using the condition of *V*_*GS*_ → ∞, i.e., 1/(*V*_*GS*_ − *V*_*T*_) → 0, because the term *L*/*μC*_*G*_ *W*(*V*_*GS*_ – *V*_*T*_) in Eq. (10) is negligible for large *V*_*GS*_ values. The plot of *R*_*total*_ vs. 1/(*V*_*GS*_ – *V*_*T*_) demonstrated that the extracted *R*_*D*_ + *R*_*S*_ was expected to be more accurate than the conventional *R*_*total*_ extrapolation from the plot for *R*_*total*_ vs. *V*_*GS*_ because of the ease of fitting to the *R*_*D*_ + *R*_*S*_ value. Moreover, the CNT-TFTs with a higher CNT density tend to have lower combined contact resistances from the S/D electrodes, i.e., *R*_*D*_ + *R*_*S*_, which is primarily attributed to an increased contact area between the CNT network film with a higher CNT density and the electrode^22^. A summary of the results of the averaged *R*_*D*_ + *R*_*S*_ from a total of 3–4 CNT-TFTs normalized to the width of the CNT network channel with different CNT densities is shown in Fig. 3e for various physical dimensions. Importantly, the components of *R*_*D*_ + *R*_*S*_ decreased with increasing CNT network density, but the values were not altered based on the physical dimensions of the network channel, thus indicating good agreement with conventional field-effect transistor theory.

**Figure 4:**
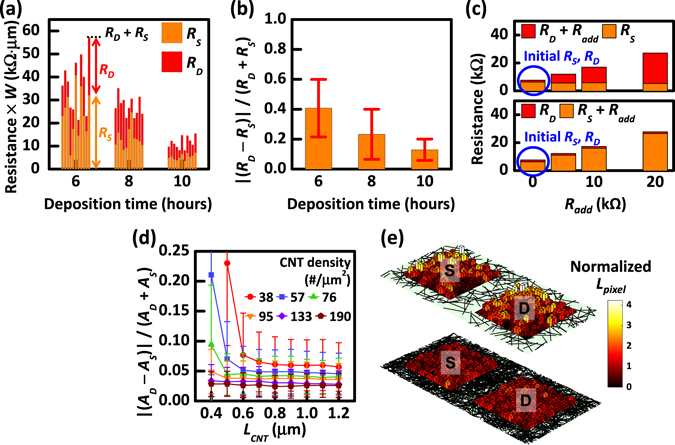
(**a**) Individual *R*_*S*_ and *R*_*D*_ values extracted from 11 CNT-TFTs for different densities of CNTs in the network. (**b**) Measured |(*R*_*D*_ − *R*_*S*_)|/(*R*_*D*_ + *R*_*S*_) of CNT-TFTs for different densities of CNTs. (**c**) *R*_*S*_ and *R*_*D*_ values of CNT-TFTs extracted with external resistance (*R*_*add*_) intentionally loaded source and drain electrodes. (**d**) Simulated asymmetric ratio of |(*A*_*D*_ − *A*_*S*_)|/(*A*_*D*_ + *A*_*S*_) of CNT-TFTs for different densities and lengths of CNTs. (**e**) Simulated distribution of contact resistance for high and low densities of CNTs in the network.

By combining the CRM and the proposed method, we finally obtained the normalized individual contact resistances of *R*_*S*_ and *R*_*D*_ in CNT-TFTs of different CNT densities in the network, as shown in Fig. 4a. The disparity of the contact interfaces with *R*_*S*_ and *R*_*D*_ at the respective S/D electrodes occurred as expected; this disparity is primarily attributed to the non-uniform contact area between networked CNTs and electrodes. For accurate analysis, we further statistically investigated the asymmetry of quantifiably extracted *R*_*S*_ and *R*_*D*_ in a total of 33 CNT-TFTs with various CNT network densities using the equation |(*R*_*D*_ − *R*_*S*_)|/(*R*_*D*_ + *R*_*S*_), defined as the asymmetry ratio, as shown in Fig. 4b. Here, a value of |(*R*_*D*_ − *R*_*S*_)|/(*R*_*D*_ + *R*_*S*_) close to 0 indicates the complete symmetry of *R*_*S*_ and *R*_*D*_. Importantly, the discrepancy between *R*_*S*_ and *R*_*D*_ decreases with increases in the CNT network density, indicating that a higher density CNT network produces higher uniformity. For the same reason, we expected that the relative standard deviation of |(*R*_*D*_ − *R*_*S*_)|/(*R*_*D*_ + *R*_*S*_), i.e., the variation of devices, would decrease with increasing CNT network density. However, we observed no clear tendency of the uniformity of CNT devices with CNT network density, as shown in the Supplementary Information, Fig. S5. However, based on the simulation in the Supplementary Information, Fig. S2, we expect that if more devices are measured, then the relative standard deviation of |(*R*_*D*_ − *R*_*S*_)|/(*R*_*D*_ + *R*_*S*_) will also decrease with increasing deposition time. As an indisputable verification of the proposed method for quantifying the individual contact interface in the CNT-TFT structure, we intentionally loaded various external resistances (*R*_*add*_ = 5, 10, and 20 kΩ) to the S/D electrodes to make the device asymmetric by design via the extrinsic resistance. As the value of *R*_*add*_ connected to the source or drain electrodes increases, the corresponding *R*_*S*_ or *R*_*D*_ were increased by the same amount of *R*_*add*_, as shown in Fig. 4c.

A comprehensive simulation was performed using the aforementioned method to further investigate and optimize the asymmetric contact interfaces in CNT-TFTs arising from the non-uniformly networked CNTs in the channel (for details of the simulation method, see the Supplementary Information, Fig. S6). First, the lengths (*L*_*CNT*_) and densities (*D*_*CNT*_) of CNTs in the network were controlled when creating the randomly networked CNT channel with an area of 3 µm × 6 µm. Next, the S/D electrodes were located at the designated 2 µm × 2 µm area. To obtain the contact area where the CNTs were in contact with the S/D electrodes, which could significantly affect the contact interface, the total lengths of CNTs were calculated underneath their respective S/D electrodes. The total lengths of CNTs at each S/D electrode are directly proportional to the contact area (*A*_*S*_ for the source electrode and *A*_*D*_ for the drain electrode) when the diameter of all CNTs simulated was assumed to be identical. Finally, the asymmetric ratio was obtained by |(*A*_*D*_ − *A*_*S*_)|/(*A*_*D*_ + *A*_*S*_) for various *D*_*CNT*_ and *L*_*CNT*_, as shown in Fig. 4d. As seen from the experimental results, the simulation results also indicate that a higher density of CNTs will yield better performance in terms of the contact interfaces, that is, nearly identical contact interfaces between the S/D electrodes, than a lower density of CNTs, as shown in Fig. 4e. For a higher density of CNTs, the distribution of contact resistances is quite uniform throughout the overall area of the electrodes, in contrast to the distribution observed for a lower density of CNTs. It is also notable that there is a sizable reduction in the asymmetric ratio when longer CNTs are utilized in the networked channel, even for a low density of CNTs. The simulation results reveal that the *L*_*CNT*_ and *D*_*CNT*_ of CNTs also play a critical role in determining the asymmetric ratio of contact interfaces at the S/D electrodes. Moreover, CNTs with a semiconducting purity above 99% should be necessary to achieve the best performance of CNT-TFTs based on the semiconducting CNTs; however, in general, the length of CNTs with such a higher semiconducting purity is shorter than those with a lower semiconducting purity, i.e., the balance of the contact interfaces at the S/D electrodes would deteriorate and be strongly dependent on *D*_*CNT*_. Therefore, for the best optimization of the performance in CNT-TFTs, a longer length and higher density of CNTs underneath the S/D electrodes is necessary in terms of the improved contact interfaces. Ultimately, our proposed method for quantifying the individual contact interfaces provides clear evidence of the need for additional studies on the contact interfaces to minimize the unbalanced contact resistances at the S/D electrodes.

In summary, we proposed a method for quantifying the contact interfaces in CNT-TFTs by directly extracting each contact resistance of *R*_*D*_ and *R*_*S*_. Each *R*_*D*_ and *R*_*S*_ was obtained using the simple measurement of transconductance values for the difference between *R*_*D*_ and *R*_*S*_, and the CRM for the sum of *R*_*D*_ and *R*_*S*_. *R*_*D*_ and *R*_*S*_ became more balanced for a high density of CNTs than for a low density of CNTs in the network in CNT-TFTs. The developed simulation was also performed while considering the randomness of the CNTs in the network to predict and optimize the performance of the CNT-TFTs. The results indicate that the length and density of the CNTs in the network can significantly affect the uniform contact interfaces; hence, a longer length and a higher density of CNTs is necessary to reduce the asymmetric contact interfaces in the S/D electrodes. Therefore, to optimize the performance of CNT-TFTs, CNTs with a semiconducting purity of above 99% should be required, and additional control of the length and density of CNTs is simultaneously indispensable. In this work, we did not focus on further accurate determination of each contact interface, including *R*_*J*_; however, *R*_*J*_ is expected to play a crucial role in the performance of CNT-TFTs with a relatively short-channel length. In these CNT-TFTs, there are few CNT-CNT junctions within the network channel, resulting in a significant contribution of *R*_*J*_ to the total resistance. Hence, further work is required to investigate the effect of *R*_*J*_ on our proposed method. Nevertheless, we believe that these findings can help minimize the impact of contact resistance on the performance and variation of CNT-TFTs and guide further development of CNT-TFTs.

## Methods

### Functionalization of Si Wafers

The cleaned wafer was immersed into a poly-L-lysine (PLL) solution (0.1 g/mL; Sigma-Aldrich) for 5 min to functionalize the SiO_2_ surface to increase the adhesion between the CNT films and the substrate^8, 23, 24^. The functionalized wafer was then rinsed with DI water to remove the unattached monomers and blown dry thoroughly using N_2_ gas^22^. Subsequently, the substrate was immersed in highly purified 99% semiconducting enriched CNT solution (concentration: 0.01 mg/ml, average diameter: 1.4 nm, average length: 1–2 µm, type of purification: density gradient ultracentrifugation, provided by NanoIntergris, Inc.).

### Characterization of CNT-TFTs

The microscopic morphology of the CNTs was determined using AFM equipment (model XE-100). The CNT-TFTs were characterized using an Agilent 4156 C semiconducting parameter analyser.

## Electronic supplementary material

Supporting Information

## References

[1] Cao Q (2008). Medium-scale carbon nanotube thin-film integrated circuits on flexible plastic substrates. Nature.

[2] Wang C (2009). Wafer-scale fabrication of separated carbon nanotube thin-film transistors for display applications. Nano Lett..

[3] Zhang J, Wang C, Zhou C (2012). Rigid/flexible transparent electronics based on separated carbon nanotube thin-film transistors and their application in display electronics. ACS Nano.

[4] Artukovic E, Kaempgen M, Hecht DS, Roth S, Grüner G (2005). Transparent and flexible carbon nanotube transistors. Nano Lett..

[5] Javey A, Guo J, Wang Q, Lundstrom M, Dai H (2003). Ballistic carbon nanotube field-effect transistors. Nature.

[6] Franklin AD (2012). Sub-10 nm carbon nanotube transistor. Nano Lett..

[7] Cao Q, Rogers JA (2009). Ultrathin films of single-walled carbon nanotubes for electronics and sensors: a review of fundamental and applied aspects. Adv. Mater..

[8] Wang C (2012). Extremely bendable, high-performance integrated circuits using semiconducting carbon nanotube networks for digital, analog, and radio-frequency applications. Nano Lett..

[9] Zhang J, Wang C, Fu Y, Che Y, Zhou C (2011). Air-stable conversion of separated carbon nanotube thin-film transistors from p-Type to n-Type using atomic layer deposition of high- κ oxide and Its application in CMOS logic circuits. ACS Nano.

[10] Sun DM (2011). Flexible high-performance carbon nanotube integrated circuits. Nat. Nanotechnol.

[11] Snow ES, Novak JP, Campbell PM, Park D (2003). Random networks of carbon nanotubes as an electronic material. Appl. Phys. Lett..

[12] Wang C, Zhang J, Zhou C (2010). Macroelectronic integrated circuits using high-performance separated carbon nanotube thin-film transistors. ACS Nano.

[13] Choi S-J, Bennett P, Lee D, Bokor J (2015). Highly uniform carbon nanotube nanomesh network transistors. Nano Res.

[14] Arnold MS, Green AA, Hulvat JF, Stupp SI, Hersam MC (2006). Sorting carbon nanotubes by electronic structure using density differentiation. Nat. Nanotechnol.

[15] Arnold MS, Stupp SI, Hersam MC (2005). Enrichment of single-walled carbon nanotubes by diameter in density gradients. Nano Lett..

[16] Tu X, Manohar S, Jagota A, Zheng M (2009). DNA sequence motifs for structure-specific recognition and separation of carbon nanotubes. Nature.

[17] Moshammer K, Hennrich F, Kappes MM (2009). Selective suspension in aqueous sodium dodecyl sulfate according to electronic structure type allows simple separation of metallic from semiconducting single-walled carbon nanotubes. Nano Res..

[18] Liu H, Nishide D, Tanaka T, Kataura H (2011). Large-scale single-chirality separation of single-wall carbon nanotubes by simple gel chromatography. Nat. Commun..

[19] Fagan JA (2014). Isolation of specific small‐diameter single‐wall carbon nanotube species via aqueous two‐phase extraction. Adv. Mater..

[20] Franklin AD, Chen Z (2010). Length scaling of carbon nanotube transistors. Nat. Nanotechnol.

[21] Franklin AD, Farmer DB, Haensch W (2014). Defining and overcoming the contact resistance challenge in scaled carbon nanotube transistors. ACS Nano.

[22] Cao C, Andrews JB, Kumar A, Franklin AD (2016). Improving contact interfaces in fully printed carbon nanotube thin-film transistors. ACS Nano.

[23] Lee D (2014). High-performance thin-film transistors produced from highly separated solution-processed carbon nanotubes. Appl. Phys. Lett..

[24] Choi S-J (2012). Comparative study of solution-processed carbon nanotube network transistors. Appl. Phys. Lett..

[25] Rouhi N, Jain D, Zand K, Burke PJ (2011). Fundamental limits on the mobility of nanotube-based semiconducting inks. Adv. Mater..

[26] Snow ES, Campbell PM, Ancona MG, Novak JP (2005). High-mobility carbon-nanotube thin-film transistors on a polymeric substrate. Appl. Phys. Lett..

[27] Wang C (2013). User-interactive electronic skin for instantaneous pressure visualization. Nat. Mater..

[28] Taur, Y. & Ning, T. H. *Fundamentals of modern VLSI devices* (Cambridge University Press, 1998).

[29] Chandra B, Park H, Maarouf A, Martyna GJ, Tulevski GS (2011). Carbon nanotube thin film transistors on flexible substrates. Appl. Phys. Lett..

