# Supplementary material for: Determination of individual contact interfaces in carbon nanotube network-based transistors
Source: Sci Rep. 2017 Jul 14;7:5453. doi: 10.1038/s41598-017-05653-x (PMC5511201; doi:10.1038/s41598-017-05653-x)
Supplement: Supplementary file 1 — Supporting Information [file 41598_2017_5653_MOESM1_ESM.doc]

Supplementary Information

**Determination of individual contact interfaces in carbon nanotube network-based transistors**

Jinsu Yoon^1,A^, Meehyun Lim^2,A^, Bongsik Choi^1^, Dong Myong Kim^1^, Dae Hwan Kim^1^, Sungho Kim^3^,*,

and Sung-Jin Choi^1^,*

^1^School of Electrical Engineering, Kookmin University, Seoul 02707, Korea

^2^Mechatronics R&D Center, Samsung Electronics, Gyeonggi-do 18448, Korea

^3^Department of Electrical Engineering, Sejong University, Seoul 05006, Korea

*Correspondence to S. J. C. & S. K. ([sjchoiee@kookmin.ac.kr](mailto:sjchoiee@kookmin.ac.kr) & [sungho85kim@sejong.ac.kr](mailto:sungho85kim@sejong.ac.kr))

^A^These authors contributed equally to this work.

**Table of contents**

1. Detailed simulation method of a randomly networked CNT film
2. Defining the uniformity of a CNT network in the simulation
3. Summary of the electrical properties of the CNT-TFTs with various channel lengths
4. Comparison of the extracted combined contact resistance (*R_D_* + *R_S_*) using two methods: the channel resistance method (CRM) and transmission line model (TLM)
5. Relative standard deviation of |(*R_D_* – *R_S_*)|/(*R_D_* + *R_S_*) with CNT network density
6. Detailed simulation method for the investigation of an asymmetric contact interface

**1. Detailed simulation method of a randomly networked CNT film**

To investigate the electrical and electromechanical properties of CNT-based nanocomposites, a 2D percolation-based model based on the Monte Carlo method was derived by randomly distributing straight 1D CNTs with a Gaussian distribution of the values of the predefined length (*L_CNT_*). We employed a 2D thin film model to reduce the computational demand.

1. **Generation of random CNT network & determining the current path**

For a specific CNT concentration (*N*) and 2D film dimensions (*L* × *W*), the CNT location was chosen by randomly distributing the centre points (*x*_0_, *y*_0_) in the 2D film. Straight 1D CNTs were generated using the *L_CNT_* and a randomly generated CNT orientation (*θ*), and their two-end points, (*x*_1_, *y*_1_) and (*x*_2_, *y*_2_), were represented in a Cartesian coordinate system.

(*x*_1_, *y*_1_) = (*x*_0_, *y*_0_) + (cos*θ*, sin*θ*)·*L_CNT_*/2 (S1)

(*x*_2_, *y*_2_) = (*x*_0_, *y*_0_) - (cos*θ*, sin*θ*)·*L_CNT_*/2 (S2)

All CNTs were identified by numbers, and their geometric information was stored in matrix form.

In the generated CNT network, the CNTs that were connected together and their junction points were identified. Assuming that all CNTs are straight, the junction position was found by simply solving sets of linear equations. All of the types of information related to each junction, including its position and the related CNT numbers, were stored in a junction matrix. We considered only a direct CNT-to-CNT intersection, i.e., direct electrical contact, in this simulation (tunnelling between neighbouring CNTs was not considered). Using the junction matrix, we could find current paths formed by the connected CNTs to the source and drain terminals. The information of all CNTs involved in the current paths was classified and stored in a new matrix.

**Figure S1-1**. Randomly generated CNT network with a concentration (*N* = 40) and a Gaussian distributed predefined CNT length (mean of *L_CNT_* = 0.7 µm) in the representative 2D area (2 µm × 2 µm). All CNTs contributing to the current path in the network are marked in red. The geometric information of each CNT is represented by the parameters of *L_CNT_*, *θ*, and (*x*_0_, *y*_0_) in a Cartesian coordinate system, as shown on the right side.

1. **Calculation of the CNT density**

The density of CNTs was defined as the number of CNTs in the unit area (1 µm × 1 µm). For a specified CNT concentration and 2D film dimension, the density (*D_CNT_*) was obtained from the average value of the CNT densities simulated by 50 runs and expressed as Eq. (S3), in which *D*_n_ is the density obtained for the n^th^ simulation run. The repetition number above 50 was unnecessary due to saturation.

*D_CNT_* = (*D*_1_ + *D*_2_ + … + *D*_n_) / n (S3)

**Figure S1-2.** Randomly distributed CNT networks for 4 runs with a concentration (*N* = 200) and Gaussian distributed predefined CNT length (mean of *L_CNT_* = 0.7 µm) in the 2D film with an area of 2 µm × 2 µm. The density for each run is specified above. The representative density (*D_CNT_*) was defined as the averaged value of all densities simulated by 50 runs.

**2. Defining the uniformity of the CNT network in the simulation**

To quantify and visualize the uniformity in the CNT network, the entire CNT network with a size of 2 µm × 2 µm was divided into a large number of small pixels with a size of 0.1 µm × 0.1 µm (total number of pixels is 400); then, we calculated two quantities for defining the uniformity of the CNT network: the total CNT length in each pixel (*L_pixel_*) and number of CNTs passing through each pixel (*N_pixel_*).

The uniformity (*σ*) was defined using the standard deviation (STD) and average (AVE) value of each quantity for all pixels according to the following formula:

*σ* = (STD/AVE) × 100 (S4)

Each quantity was then normalized based on the averaged value for all pixels in the entire CNT network. Based on the simulation result, the uniformity of CNT networks exponentially decreased, i.e., improved, with density in the range applied (Fig. S2).

**Figure S2.** Calculated uniformity of the CNT networks at different densities for two quantities, *L_pixel_* and *N_pixel_*, and their 2D images represented by 400 pixels with a pixel size of 0.1 µm × 0.1 µm.

**3. Summary of the electrical properties of the CNT-TFTs with various channel lengths**

The key device performance metrics, such as log (*I_ON_*/*I_OFF_*), normalized on-state current (-*I_ON_*/*W*), threshold voltage (*V_T_*), normalized transconductance (*g_m_*/*W*), and field-effect mobility (*µ*), with CNT deposition time (6, 8, and 10 h) are summarized in Fig. S3. The CNT-TFTs with larger channel lengths (*L*) tend to have higher log (*I_ON_*/*I_OFF_*), which results from the lower probability of metallic CNTs to form percolating pathways between S/D in long-channel CNT-TFTs. However, as the CNT deposition time increases, the tendency of log (*I_ON_*/*I_OFF_*) with *L* gradually disappears because there are many metallic paths regardless of *L*. Additionally, both -*I_ON_*/*W* and *g_m_*/*W* are approximately proportional to the reciprocal of *L* (1/*L*), in agreement with conventional field-effect transistor operation theory. In addition, *µ* seems to decrease with decreasing *L* because the current is more limited by contact resistance with decreasing *L*.

**Figure S3.** Log(*I_ON_*/*I_OFF_*), normalized on-state current (-*I_ON_*/*W*), threshold voltage (*V_T_*), normalized transconductance (*g_m_*/*W*), and mobility (*µ*) for the CNT-TFTs with different *L*: (a) deposition time of 6 h, (b) deposition time of 8 h, and (c) deposition time of 10 h.

**4. Comparison of the extracted combined contact resistance (*R_D_* + *R_S_*) using two methods: the channel resistance method (CRM) and transmission line model (TLM)**

We also extracted the combined contact resistances (*R_D_* + *R_S_*) using the TLM from the *I_DS_*-*V_GS_* measurements (for *V_DS_* = -0.5 V) of the CNT-TFTs with different *L* values (*L* = 1.4, 2.2, and 3.0 µm) at different *V_GS_* – *V_T_* values, as shown in Fig. S4a. For each value of *V_GS_* – *V_T_*, the *V_DS_*/*I_DS_* ratio, i.e., the total resistance *R_total_*, increased linearly with increasing *L*, with the cross point of linear regressions indicating *R_D_* + *R_S_*^1^. We obtained *R_D_* + *R_S_* values in the CNT-TFTs produced with different deposition times (6, 8, and 10 h), as shown in Fig. S4b. The *R_D_* + *R_S_* values extracted using the CRM are also shown in the figure. The *R_D_* + *R_S_* values extracted using the CRM and TLM were very similar.

Each resistance value in Fig. S4b was normalized with *W*. Using the CRM, *R_D_* + *R_S_* values can be extracted from all CNT-TFTs; hence, the values were averaged and are shown with the standard deviation. However, for the TLM, only one value of *R_D_* + *R_S_* can be obtained from the CNT-TFTs with different *L* values.

**Figure S4.** (a) Extraction of the combined contact resistances (*R_D_* + *R_S_*) of the CNT-TFTs produced from a deposition time of 6 h using the TLM. (b) Comparison of the extracted normalized *R_D_* + *R_S_* using the CRM and TLM.

**5. Relative standard deviation of |(*R_D_* – *R_S_*)|/(*R_D_* + *R_S_*) with CNT network density**

To investigate the uniformity of the CNT network, we calculated the relative standard deviation of |(*R_D_* – *R_S_*)|/(*R_D_* + *R_S_*) with CNT network density using the standard deviation (STD) and average (AVE) value according to the following formula:

Relative standard deviation = (STD/AVE) (S5)

We confirmed that there was no clear tendency of the uniformity of the CNT device with CNT network density as shown in Fig. S5. However, we expect that if more devices are measured, the relative standard deviation of |(*R_D_* – *R_S_*)|/(*R_D_* + *R_S_*) will decrease with increasing deposition time based on the results of simulation (Fig. S2).

**Figure S5.** Relative standard deviation of |(*R_D_* – *R_S_*)|/(*R_D_* + *R_S_*).

**6. Detailed simulation method for investigating the asymmetric contact interface**

To investigate the asymmetric contact interface in this simulation, we considered the CNT network with a total area of 3 µm × 6 µm with S/D electrodes located in the designated 2 µm × 2 µm area, as shown in Fig. S6. The contact resistance between the CNTs and each electrode is approximately inversely proportion to the size of the contact area in which the CNTs were contacted to the S/D electrodes. We assumed that the diameters of all CNTs simulated are identical. Therefore, the contact asymmetry was estimated from the asymmetry of the total CNT lengths at the S/D electrodes calculated in the simulation, expressed as Eq. (S6). We considered only CNTs forming current paths between the S/D electrodes.

|(*R_D_* – *R_S_*)|/(*R_D_* + *R_S_*) ≈ |(*A_D_* – *A_S_*)|/(*A_D_* + *A_S_*) = |(*L_D_* – *L_S_*)|/(*L_D_* + *L_S_*) (S6)

**Figure S6.** Configuration of the CNT network to investigate the asymmetric contact interface for *D_CNT_* = 95 #/µm^2^ and a mean of *L_CNT_* = 0.7 µm.

**Supplementary Information References**

1. Taur, Y. & Ning, T. H. *Fundamentals of modern VLSI devices* (Cambridge University Press, 1998).
